# Integration of Consonant and Pitch Processing as Revealed by the Absence of Additivity in Mismatch Negativity

**DOI:** 10.1371/journal.pone.0038289

**Published:** 2012-05-31

**Authors:** Shan Gao, Jiehui Hu, Diankun Gong, Sifan Chen, Keith M. Kendrick, Dezhong Yao

**Affiliations:** 1 Key Laboratory for NeuroInformation of Ministry of Education, School of Life Science and Technology, University of Electronic Science and Technology of China, Chengdu, China; 2 School of Foreign Languages, University of Electronic Science and Technology of China, Chengdu, China; 3 Mental Health Education and Counseling Center, University of Electronic Science and Technology of China, Chengdu, China; Baycrest Hospital, Canada

## Abstract

Consonants, unlike vowels, are thought to be speech specific and therefore no interactions would be expected between consonants and pitch, a basic element for musical tones. The present study used an electrophysiological approach to investigate whether, contrary to this view, there is integrative processing of consonants and pitch by measuring additivity of changes in the mismatch negativity (MMN) of evoked potentials. The MMN is elicited by discriminable variations occurring in a sequence of repetitive, homogeneous sounds. In the experiment, event-related potentials (ERPs) were recorded while participants heard frequently sung consonant-vowel syllables and rare stimuli deviating in either consonant identity only, pitch only, or in both dimensions. Every type of deviation elicited a reliable MMN. As expected, the two single-deviant MMNs had similar amplitudes, but that of the double-deviant MMN was also not significantly different from them. This absence of additivity in the double-deviant MMN suggests that consonant and pitch variations are processed, at least at a pre-attentive level, in an integrated rather than independent way. Domain-specificity of consonants may depend on higher-level processes in the hierarchy of speech perception.

## Introduction

The relationship between language and music has been a matter of controversy for years. Comparisons have involved a number of basic processes in these two systems, such as syntax [1], [2], meaning [3], [4] and rhythm [5]. In contrast to the large amount of research on these processes within the two domains, less exploration of fundamental elements shared by them (e.g., phoneme vs. pitch) has been carried out.

Bigand, Tillmann, Poulin, D'Adamo, & Madurell [6] measured the harmonic priming effect on phoneme monitoring in vocal music and found an interaction at some stage of processing between phonology and harmonic congruity. They manipulated the structural relationship between the last sung chord and the preceding musical context, an eight-chord sung sequence, and found faster phoneme monitoring of the last sung vowel when it was sung on the tonic (or congruent) than on the subdominant chord. However, Kolinsky, Lidji, Peretz, Besson, & Morais [7] argued that Bigand et al.'s finding [6] might not generalize to musical and phonemic processing as a rule, since in that study only one phoneme category was adopted for discrimination, namely vowels (i.e., the /di/-/du/ distinction) and vowels differ from the other phoneme category (i.e., consonants) in both acoustical property and linguistic function.

Using a speeded classification paradigm [8] of bi-syllabic non-words sung on two-note melodic intervals, Kolinsky et al. [7] examined the interference between pitch processing and that of either vowels or consonants. They observed that vowel and pitch dimensions cannot be attended selectively. When the non-word classification was based on vowel identity, irrelevant variations in pitch interfered with the classification process. A similar interference of irrelevant vowel changes was found when the classification was based on pitch. In contrast, there was weaker interference between pitch and consonant manipulations. These findings seem to suggest that vowels are processed in a more integrated way than consonants. Further, Lidji et al. [9] demonstrated early, pre-attentive interactions between vowels and pitch when sung, which was indexed electrophysiologically by the additivity of the mismatch negativity (MMN) in evoked potentials.

The (auditory) MMN is a fronto-centrally negative event-related potential (ERP) component originally found in the oddball paradigm where infrequent (deviant) stimuli are presented among frequent (standard) stimuli. The MMN is elicited when a deviant acoustically differs from a repeated standard sound in a stimulus dimension such as frequency, intensity or duration. It usually peaks between 100 and 250 ms after the onset of the deviant. The measurement of this component has been often applied to tap into the initial stages of auditory processing. Occurring generally in conditions of inattention, the MMN is typically argued to reflect pre-attentive detection of auditory deviations [10], [11]. It is mainly generated in the auditory cortex in the temporal lobes, but may also be contributed to by other brain structures. For example, some evidence suggests the contribution of the frontal activation, which might be associated with the involuntary switching of attention to changes appearing outside the focus of attention [12], [13]. Indeed, attentional modulation on MMN responses has been reported in several studies although a number of different interpretations on the specific MMN generator mechanism have been proposed [14].

The MMN additivity approach in this context has the following logic: if the deviations of two sound dimensions are processed by separate neural generators, then the amplitude of the MMN response to a bi-dimensional deviation should equal the sum of the corresponding uni-dimensional MMNs. Indeed, previous studies have found the MMNs to double deviants to be additive for several dimension conjunctions in both simple tone stimuli [15]–[17] and complex speech sounds [18]. Conversely, if the two stimulus features are processed by common or interactive sources, the double deviants will elicit a MMN smaller than the sum of the MMNs in response to the corresponding single stimulus features [19]. This under-additivity pattern has been observed for the combination of frequency and intensity [17]. Lidji et al. [9] utilized this MMN additivity approach to investigate the independent or integrated early pre-attentive neural processes of vowels and pitch height. Results showed under-additivity of the uni-dimensional MMN responses, indicating vowels and pitch are processed by interactive neural networks.

Lidji et al. [9] provided electrophysiological evidence for part of the findings of Kolinsky et al. [7]—the vowel-pitch interaction arising from relatively late cognitive processes in a behavioral task. However, the separability of consonants and pitch, which was also found by Kolinsky and collaborators [7], has not yet been measured using the approach of MMN additivity. It is possible that consonants, though regarded as more speech-specific [7], share some more general neural sources with pitch at pre-attentive processing stages before, or even during processing at a phonetic level. Previous research [20], [21] has proposed that phonetic perception involves two distinct levels of processing: an auditory level and a phonetic level. Although unidirectional or asymmetric, an interaction between phonetic and auditory dimensions of sound stimuli (i.e., /bae/−l04 Hz, /bae/−140 Hz, /gae/−l04 Hz, and /gae/−140 Hz) has been observed by Wood [22], indicating that the component processes for phonetic information are in some way dependent upon those for auditory information. Moreover, some fMRI studies [23] have suggested that there are functional hierarchies within the auditory cortex, such that lower levels of processing are dependent upon bilateral core areas, whereas there has also been a lot of discussion on distinct neural substrates between pitch and phoneme processing [24].

In the current study we have therefore attempted to resolve the issue of whether consonants and pitch are processed by separate or common neural substrates in the auditory system at lower levels when speech may be more accurately characterized as acoustic rather than linguistic in nature. In order to establish the presence or absence of such integrated processing of consonants and pitch, we have investigated whether the MMN responses to consonant and pitch features are additive or not when presented as double deviants. Results found no evidence for an additivity pattern in the MMN providing the first experimental support for the integration of consonant and pitch processing at a pre-attentive level.

## Materials and Methods

### Participants

Twelve paid volunteers (mean age 24.5 years, range: 22–34) participated in this experiment. All of them were right-handed males who were native Chinese speakers and reported no history of hearing deficits or any brain impairment. None of them had received formal musical training. Written consent was obtained from all participants before the experiment, and the experiment was approved by the review board of the Key Laboratory for NeuroInformation of Ministry of Education in University of Electronic Science and Technology of China.

### Stimuli

Stimuli were Chinese initial consonants /t/ and /k/ (actually pronounced as consonant-vowel syllables /te/ and /ke/ when alone). They were sung at two pitches (C3 = 130 Hz and D3 = 146 Hz; see Table 1) with a duration of 300 ms. A baritone was chosen to sing the syllables in order to avoid substantial phoneme distortions due to high frequencies generally observed in female opera singers [25], [26]. The choice of this frequency distance was based on the results of calibration pilot studies which showed that the MMN amplitude for the one tone discrepancy matched the amplitude of the MMN to the /t/-/k/ contrast. The acoustic properties of the sounds were analyzed with Praat software; frequency, intensity, consonant and vowel duration of the four stimuli were normalized with Adobe Audition software (see Figure S1 for spectrograms). After normalization, voice onset time of the same category of consonants varied by less than 3 ms. To avoid phoneme distortions, stimuli matched for acoustic parameters were selected out of a large number of recordings before normalization.

**Table 1:** Fundamental frequency (F0) and frequency of the three first formants (F1, F2, F3) for the sung consonants in Hz.

Stimulus	/t/ C3	/k/ C3	/t/ D3	/k/ D3
F0	130	130	146	146
F1	464	503	476	511
F2	1103	1123	1129	1130
F3	2609	2657	2636	2666

### Procedure

The sound stimuli were presented using E-prime II software at an offset to onset interval of 500 ms in a sequence consisting of frequent (standard) and infrequent (deviant) stimuli. The presentation was pseudo-randomized so that any two deviants were separated by no less than three standards. The deviants were different from the standards in three ways: consonant identity only, pitch height only, or both consonant identity and pitch height. With a mixed design [18], all the three deviant types occurred in each of the four blocks of stimulus presentation. Each of the four stimuli (/t/-C3, /k/-C3, /t/-D3, /k/-D3) served as the standard, and the other three as deviants across blocks. The presentation order of the four blocks was counterbalanced across participants and each block lasted for 16 min. In the whole session, 4760 sounds were presented to each participant, including a total of 280 occurrences of each type of deviant (probability of occurrence = .06) and 3920 standards. During the experiment, the participants were watching a silent self-selected subtitled movie in an electrically and acoustically isolated room. They were instructed to focus on the movie and to ignore the auditory stimulation presented binaurally through headphones at an intensity level of 70 dB SPL.

### EEG Recording

The EEG (bandpass 0.01–100 Hz, sampling rate 500 Hz) was recorded with a cap of 64 Ag-AgCl electrodes connected according to the extended 10–20 system. The impedance for all electrodes was kept below 10 kΩ. All channels were measured with frontal vertex (i.e., FCz) as the reference and converted to a linked-mastoid reference off-line. AFz served as the ground electrode during recording. Participants were asked to avoid eye blinking, to stay still and to relax their facial muscles. To control for eye movement artifacts, horizontal and vertical EOG were monitored by electrodes respectively placed above the left eye and at the outer canthus of the right eye.

Off-line analysis was performed with the computer software Brain Vision Analyzer Version 2.0.1 (Brain Products GmbH). The EEG data were further filtered (0.01–30 Hz) and corrected for ocular artifacts. Then, recordings were re-referenced to “infinity” by the reference electrode standardization technique (REST) off-line (Free software download at doi:10.1016/j.clinph.2010.03.056) [27]. REST is an equivalent distributed source based computer algorithm which translates practical recordings with non-zero reference such as linked-ears or average reference to a reference at infinity where the potential is zero. It therefore provides a method to remove non-zero or active reference effects from the recordings [28]–[30].

Artifacts exceeding ±100 µV were also discarded but the number of such trials did not exceed 25% in a single block. Before segmentation the standard sounds preceded by a deviant were excluded from further analyses in case they might have evoked an MMN-like response. Epochs of 700 ms, including a 200 ms pre-stimulus interval for baseline correction, were averaged separately for stimulus identity (/t/-C3, /k/-C3, /t/-D3, /k/-D3) when playing the role of either standard or deviant across blocks. Epochs of stimulus category (standard, consonant deviant, pitch deviant, double deviant) were also averaged across the four different stimuli, which allowed us to generalize the results to distinct consonants and pitches, thus avoiding stimulus specific effects.

### Computation of ERPs

First of all, in order to examine whether MMN amplitude and latency were modulated by stimulus identity, the MMN for each stimulus was extracted individually by subtracting the waveform to this stimulus when used as a standard from the one when it was used as a consonant deviant, as a pitch deviant and as a double deviant, employing the flip-flop method [31]. The MMN mean amplitude was calculated as the mean voltage at an 80-ms period centered on each individual's peak detected 100–250 ms after stimulus onset. The peak latency was defined as the time point of the maximum negativity in the same time window. Secondly, the MMN independent of stimulus identity was delineated by subtracting the waveform to the standard averaged across all four different stimuli when playing this role, from the similarly computed waveform for each type of deviant. This yielded three MMNs: consonant deviant, pitch deviant, and double deviant. In these waves, MMN amplitude and latency were measured as for the stimulus-specific MMNs.

To test the MMN additivity hypothesis, the mean amplitude of the empirical double deviant MMN was individually compared to that of the predicted double deviant MMN, which was obtained as the sum of the consonant and the pitch deviant stimulus-independent difference waves. The mean amplitudes of the observed and predicted double deviant MMNs were quantified over a 80-ms period in the same way as the time window chosen for the uni-dimensional MMNs.

The fronto-central electrode (Fz) was chosen for the main analyses because the MMN amplitude was generally maximal at this site. However, in consideration of the possibility that individual differences in amplitude distribution might contribute to the results, a cluster of electrodes (Fz, F1, F2, FCz, FC1, FC2) was also analyzed. The results were very similar to those using a single Fz (see Results S1). Therefore, only the results obtained at Fz are presented below. Differences between the presentation conditions were analyzed using ANOVAs and, the Greenhouse-Geisser correction for non-sphericity was applied when required and the corrected *p* is reported along with the original degrees of freedom.

## Results

### Effects on the MMN response

The presence of the MMN for each stimulus and each type of deviant was confirmed at an individual level with two-tailed *t*-tests comparing the mean amplitude of the difference wave at Fz with the average baseline level (i.e., zero). The MMNs were significant with *t*-values ranging between −2.482 and −59.684 and with *p*<.017 in all cases.

**Figure 1:**
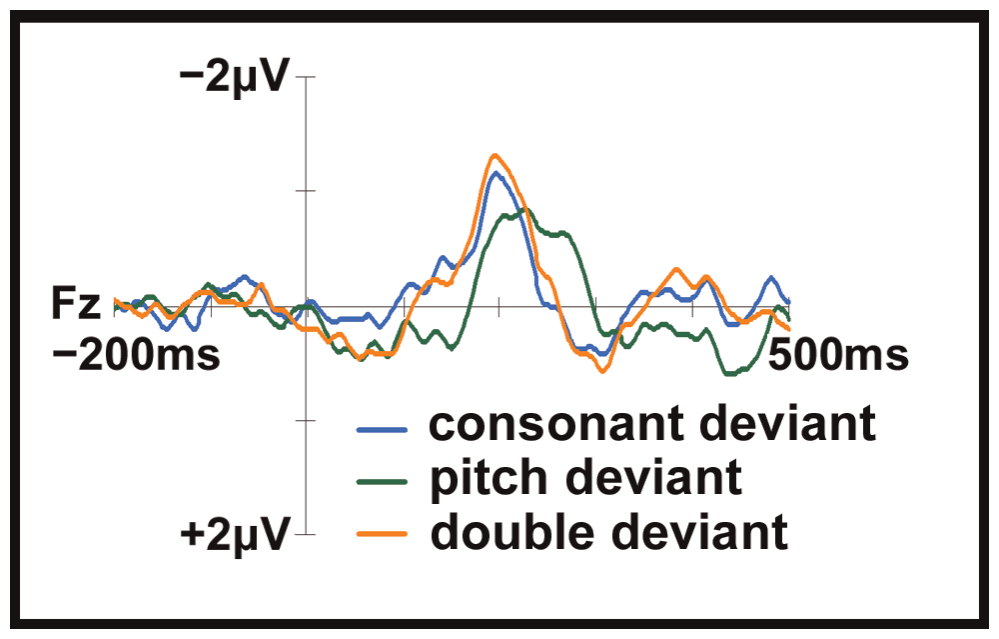
The deviant – standard difference waveforms averaged across the four stimuli. The double deviant, −1.1±0.3 µV, *t*(11) = −10.938, as well as the consonant, −0.9±0.4 µV, *t*(11) = −7.428, and the pitch deviant, −0.9±0.4 µV, *t*(11) = −7.662, elicited a significant MMN at Fz.

In order to analyze potential effects of the physical identity of the stimulus, a two-way analysis of variance was conducted on MMN mean amplitude and peak latency at Fz, with stimulus identity and deviant type as within-subjects variables. The mean amplitudes and latencies of the MMNs did not differ significantly between stimuli, *F*<1.8, *p*>.18 in both cases. The interaction between stimulus identity and deviant type was also not significant for either amplitude, *F*(6, 66) = 1.613, *p* = .15, or for latency, *F*(6, 66) = 1.005, *p* = .43. In short, stimulus identity did not modulate MMN amplitude or latency and did not interact with the type of deviant. Accordingly, the MMN for each type of deviant could be delineated based on the averaged ERPs across the four physically different stimuli (see Figure 1). In this way, we concentrated on the deviant types rather than incidental physical dissimilarities across stimuli.

Separate one-way within-subjects ANOVAs were conducted at Fz so as to examine whether MMN amplitude and latency differed as a function of deviant type. The MMN amplitudes for each category of deviant were not significantly different from one another, *F*(2, 22) = 2.010, *p* = .158. On the other hand, MMN latencies were significantly influenced by deviant type, *F*(2, 22) = 6.448, *p* = .006 (see Figure 1 and Table 2). Post-hoc comparisons with a Bonferroni adjustment revealed that the pitch deviation elicited a later MMN peak (224±25 ms) than did the consonant (202±25 ms, *F*(1, 11) = 8.189, *p* = .015) and the double (197±26 ms, *F*(1, 11) = 13.66, *p* = .004) deviants.

**Table 2:** Different types of the MMN and the MMN additivity test.

Type of the MMN	Mean Amplitude	SD
Consonant single deviant MMN	−.8586	.4004
Pitch single deviant MMN	−.8576	.3878
Observed double deviant MMN	−1.0808†	.3423
Predicted double deviant MMN*	−1.5541‡	.5955

* The predicted double deviant MMN was computed for each subject as the sum of the consonant single deviant and the pitch single deviant MMNs at Fz.

† There was no significant difference between the observed double MMN and the two single MMNs (*p* = .158).

‡ The predicted double MMN was smaller than the observed double MMN (*p* = .003, paired-sample *t*-test).

For the purpose of measuring the diversities among the topographies of brain responses to consonant, pitch, and double changes (see Figure S2), a two-way repeated measures ANOVA was conducted on the MMN mean amplitudes, with deviant (3 levels) and electrode (61 levels) as within-subject variables. As expected, we found a main effect of electrode, *F*(60, 660) = 9.206, *p*<.0001, but no interaction between electrode and type of deviant, *F*(120, 1320) = 1.004, *p* = .473, showing that the scalp distribution of the MMN was not different for consonant, pitch, and double deviants.

Additionally, we tested whether there were effects of laterality on the MMN distributions. We divided the anterior scalp region into two sections (left vs. right, midline excluded). Only the electrodes in the frontal and central areas, where the MMN was the largest, were pooled here, encompassing FP1, AF3, F1, F3, F5, F7, FT7, FC5, FC3, FC1, C1, C5, and T7, for the left side of the head, and FP2, AF4, F2, F4, F6, F8, FT8, FC6, FC4, FC2, C2, C6, and T8, for the right side of the head. A two-way analysis of variance on the mean, normalized MMN amplitudes of these electrodes, with laterality and deviant type as within-subject variables, demonstrated that the MMNs did not differ between the two hemispheres, *F*(1, 11) = .135, *p* = .72. No interaction between deviant type and laterality was found, *F*(38, 418) = 1.197, *p* = .202.

**Figure 2:**
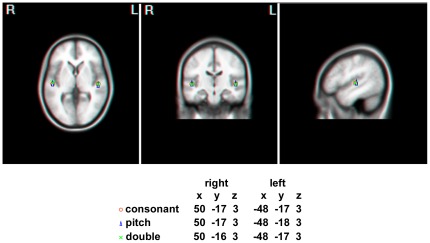
Axial, coronal, and sagittal views of grand-average dipole locations of the MMNs. Bilaterally located in the superior temporal gyri, the MMN source locations were almost identical in response to the consonant, the pitch, and the double deviances. Dipole locations are indicated with respect to the Talairach coordinate system.

A two-dipole model was also computed using EMSE software to localize the neural sources of the MMNs to the three types of deviances. Dipole locations and strengths were obtained from individual subject. The grand-average dipole sources were bilaterally located in the superior temporal gyri, which is consistent with the previous source localization results of pitch and consonant activities [32], [33], and the MMN source locations were almost identical in response to the consonant, the pitch, and the double deviances (Figure 2). To test whether the source configurations were statistically different for the different types of deviants, a two-way ANOVA was performed on the dipole strengths, with hemisphere and deviant as within-subject factors. The results found no main effects of hemisphere, *F*(1, 11) = 1.778, *p* = .209, and deviant, *F*(2, 22) = 3.188, *p* = .061, and no hemisphere×deviant interaction, *F*(2, 22) = .423, *p* = .66, indicating that there were no significant differences in MMN origins in the different conditions.

### MMN Additivity

**Figure 3:**
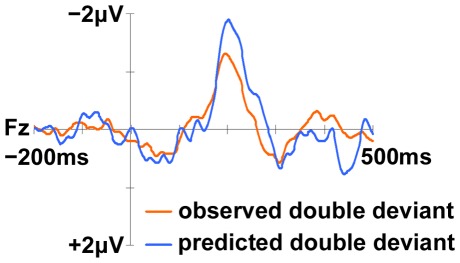
The MMNs to the observed and the predicted double deviant at Fz. The observed MMN (gray line) was significantly smaller than the predicted MMN (black line), *t*(11) = 3.884, *p* = .003, indicating the single consonant and pitch deviant MMNs were underadditive.

Although we found no evidence for significant differences between the single and double deviant MMNs, we also carried out a further analysis to support the absence of additivity. We summed the mean amplitudes of the two single deviant MMNs in order to provide a predicted double deviant MMN in the case of additivity (see Table 2). Figure 3 shows the observed and predicted double deviant difference waves at Fz. Paired-sample *t*-tests were employed to compare the two bi-dimensional MMNs at Fz. This confirmed that the empirical double deviant MMN was indeed significantly smaller than the predicted MMN, *t*(11) = 3.884, *p* = .003, again suggesting the consonant and pitch single deviant MMNs were not additive.

The absence of additivity might have resulted from the 22 ms latency difference between the consonant and pitch MMNs since the earlier processing of the consonant deviation might prevent complete processing of the pitch deviation in the bi-dimensional deviants. If so, subjects without, or with only small, latency discrepancies should display more additivity than those with larger latency discrepancies. To test for this possibility subjects were divided into two groups based on whether they showed significantly later MMN responses at Fz to pitch deviance or not. This created a significant-difference group of 5 subjects (average difference 50 ms) and an non-significant-difference group of 7 subjects (average difference 2 ms). A 2 (Latency difference: significant & non-significant)×2 (Mean amplitude: the observed MMN & the predicted MMN) ANOVA was then conducted. Results confirmed the smaller amplitude of the observed double MMN compared with the predicted one, *F*(1, 10) = 13.205, *p* = .005. There was no significant group effect, *F*(1, 10) = 1.094, *p* = .32. Importantly, the amplitude×group interaction was also not significant, *F*(1, 10) = .645, *p* = .441, indicating that the latency difference between the consonant and pitch MMNs did not modulate the degree of under-additivity and therefore could not serve as an explanation for our finding.

### Behavioral control experiment

The MMN response to pitch deviants occurred significantly later than those to consonant and to double deviants. In order to examine whether this latency discrepancy was related to the fact that pitch deviations were harder to detect than the other two, a control behavioral experiment was run.

Fifteen paid non-musicians (mean age 24, range 22–28, 2 females), 10 of whom had participated in the original MMN experiment, were required to press a key (i.e., 0) as quickly as possible when they detected a deviant in a stream of repeated sounds. The stimuli and design were exactly the same as in the MMN experiment. Subject reaction times and accuracy rates were recorded. Reaction times were significantly different between the types of deviants, F(2, 28) = 55.935, *p*<.001; as were accuracy rates, F(2, 28) = 11.437, *p*<.001. Participants were slower in detecting pitch (478 ms) compared with consonant (436 ms) and double changes (430 ms), *p*<.001 in both cases. More importantly, their detection was less accurate for pitch (mean±SD = 97±3.4%) than for consonant (98.7±2.5%) and double deviances (99.4%±1.3%), *p* at least <.004. No differences were observed between the consonant and double deviances for both reaction times, F(1, 14) = 2.265, *p* = .155, and accuracy, F(1, 14) = 3.327, *p* = .09. These results are consistent with the MMN latency difference and suggest that the pitch variation was less salient than the consonant one.

## Discussion

The present MMN study probed the relations between pre-attentive consonant and pitch processing with an oddball paradigm, where deviant sung consonant-vowel syllables were occasionally inserted in a repetitive sequence of more frequent sung syllables. The stimuli changed in either consonant identity only, pitch only, or both dimensions. Every type of deviation elicited a reliable and similar amplitude MMN at Fz. The MMN in response to the double deviance was not twice as large as the one to each single deviance. In order to confirm the under-additivity, however, a comparison was conducted between the MMN response to the bi-dimensional deviants and the sum of those to the corresponding uni-dimensional ones (i.e., the predicted double deviant MMN) and this confirmed that the observed double MMN was significantly smaller than the predicted double MMN at Fz. In consideration of the possibility that individual differences in amplitude distribution might contribute to the results, a cluster of electrodes (Fz, F1, F2, FCz, FC1, FC2) was also analyzed. The results were compatible with those obtained at single Fz (see Results S1).

However, one could argue that the latency discrepancy between the consonant and pitch MMNs might mask the additivity in the bi-dimensional condition. Lidji et al. [9] obtained a similar finding of latency discrepancy between vowel and pitch MMNs. It seemed that the pitch changes were harder to detect than the phoneme changes, as supported by a behavioral control study revealing slower and less accurate responses to the pitch deviants than to the consonant deviants Like Lidji and collaborators, we directly examined the potential influence of MMN latency discrepancies in our data. Participants with a significant latency discrepancy (50 ms on average) between consonant and pitch showed a similar non-additive pattern to those exhibiting no or a negligible latency discrepancy (2 ms on average). Czigler & Winkler's [34] also excluded latency effects and reported that the processing of frequency and duration were not additive even with an average latency discrepancy of 75 ms. In the present study, the average peak latency discrepancy between the consonant and pitch MMNs was only 22 ms. On the other hand, Levänen et al. [15] found evidence for statistical additivity in the MMN with a slightly longer average latency difference of 30 ms. Thus, the under-additivity of the one-feature deviant MMNs observed in our study could not be attributed to the small latency difference found. Instead, interactive processing seems to contribute to the non-additivity pattern in the detection of consonant and pitch variations in sung syllables. In other words, the processing of the consonant dimension is integrated with that for pitch in complex auditory stimuli at a psychoacoustic level. Since the MMN appears to be an extremely sensitive electrophysiological index of minimal acoustic differences in speech stimuli [35], the under-additivity observed in the present study may result from the general neural sources responsible for the lower-level perceptual input stage that is relevant for both speech and music.

One might also argue that there might be subtle detailed source-configuration differences for the complex auditory feature combinations used in the present study and this might result in different field-potential cancellation effects due to the local cortical convexity, which then confound the observable amplitude of the MMN in response to the double deviation. However, the MMN additivity is not just observed in experiments comparing rudimentary and orthogonal feature conjunctions like frequency and duration. Using similarly complex speech sounds, previous research has observed additivity of the MMNs in response to component features [18]. Alternatively, it is possible that there are two separate but adjacent neural populations, which process the two sound features independently but are mutually affected by lateral inhibition [36]. This could also make the combined MMN smaller than the sum of the separate MMNs to each deviance. Nevertheless, our current dipole analysis found no significant differences in the origins of different MMNs, providing further support for the integration of consonant and pitch information in the auditory stream. In the future, detailed multimodal imaging techniques, such as EEG-fMRI information fusion may provide further evidence [37], [38].

The presence of common neural mechanisms for consonant and pitch processing in the current MMN study does not however deny language-specificity documented in an established literature [24], [39]–[42]. On the contrary, this finding complements classical domain-specificity theories by suggesting that consonant processing specificity is likely to occur at more complex levels as pitch processing specificity. The processing of isolated pitches is not specific to music, but the tonal encoding of pitch might be [43]. Similarly, neither physical properties nor phoneme status of a sound are sufficient for language laterality [44]. Speech perception involves a hierarchy of processing stages: auditory, phonetic, phonological, syntactic, and semantic [45], [46]. Language specificity arises when sounds are processed at more advanced and linguistic stages (e.g., semantic). Nevertheless, at primitive stages (at least the auditory stage), speech and music properties undergo some common peripheral processing and brain imaging data lend support to this proposal. Early stages of speech processing rely on core areas bilaterally while higher-level processing mechanisms are associated with more specialized regions in the left hemisphere [23], [47]. Vouloumanos et al. [42] identified overlapping neural substrates performing complex speech and non-speech operations at an early processing stage though they emphasized some degree of functional specialization for speech. Notably, in line with these studies we found no evidence for hemispheric dominance, or regional differences, in the analyses of MMN topographies across the three types of deviants Our findings therefore provide no support for the possibility that the evoked potential signals in response to consonant and pitch changes were produced by different generators.

At first glance, our observation of a non-additive pattern in the consonant and pitch MMNs seems unexpected on account of Kolinsky et al's [7] claim that consonants do not interact with intervals during song processing. However, Kolinsky et al. [7] used Garner's interference paradigm, which does not allow one to specify the processing level of dimensional interactions. It is hence possible that weakened interactions between consonants and pitch may emerge at “higher” stages in the hierarchical model of human auditory processing to produce the separability reported in their study whereas the stronger integration of consonants and pitch observed in our study is limited to relatively early processing stages.

In summary, our electrophysiological findings have revealed the first evidence for a pre-attentive integration of consonant and pitch processing in sung stimuli. This provides further support for overlap processing between language and music. It is important for future research however, that other methodologies are employed to study this integrality of different component features of complex sounds and to confirm that cortical specializations for speech and pitch go beyond the classical dichotomies.

## Supporting Information

Results S1 Supplementary results.
(DOC)

Figure S1 **Spectrograms of the four auditory stimuli.** These sounds were Chinese initial consonants /t/ and /k/ (actually pronounced as consonant-vowel syllables /te/ and /ke/ when alone) sung at two pitches (C3 = 130 Hz and D3 = 146 Hz) with a duration of 300 ms.
(TIF)

Figure S2 **The average scalp topographies of the MMN in each condition.** The mean amplitudes of the MMNs were calculated over a 80-ms window centered at the peak. No significant difference or hemispheric dominance was found in the scalp distributions of the MMNs to consonant, pitch, and double deviants.
(TIF)
