# Supplementary material for: Integration of Consonant and Pitch Processing as Revealed by the Absence of Additivity in Mismatch Negativity
Source: PLoS One. 2012 May 31;7(5):e38289. doi: 10.1371/journal.pone.0038289 (PMC3365020; doi:10.1371/journal.pone.0038289)
Supplement: Results S1 — Supplementary results. (DOC) [file pone.0038289.s001.doc]

**Supplementary Results**

**Effects on the MMN response**

The presence of the MMN for each stimulus and each type of deviant was confirmed at an individual level with two-tailed *t*-tests comparing the mean amplitude of the difference waves averaged across a cluster of electrodes (Fz, F1, F2, FCz, FC1, FC2) with the average baseline level (i.e., zero). The MMNs were significant with *t*-values ranging between -2.997 and -58.123 and with *p* < .01 in all cases.

In order to analyze potential effects of the physical identity of the stimulus, a two-way analysis of variance was conducted on MMN mean amplitude and peak latency at the clustered electrodes, with stimulus identity and deviant type as within-subjects variables. The mean amplitudes and latencies of the MMNs did not differ significantly between stimuli, *F* < 1.7, *p* > .17 in both cases. The interaction between stimulus identity and deviant type was also not significant for either amplitude, *F*(6, 66) = .824, *p* = .556, or for latency, *F*(6, 66) = 1.108, *p* = .367. In short, stimulus identity did not modulate MMN amplitude or latency and did not interact with the type of deviant.

Separate one-way within-subjects ANOVAs were conducted on the averaged difference waveforms of the six chosen electrodes so as to examine whether MMN amplitude and latency differed as a function of deviant type. The MMN amplitudes for each category of deviant were not significantly different from one another, *F*(2, 22) = 2.182, *p* = .137. On the other hand, MMN latencies were significantly influenced by deviant type, *F*(2, 22) = 6.404, *p* = .006. Post-hoc comparisons with a Bonferroni adjustment revealed that the pitch deviation elicited a later MMN peak at Fz (225 ± 24 ms) than did the consonant (201 ± 25 ms, *F*(1, 11) = 7.923, *p* =.017) and the double (198 ± 26 ms, *F*(1, 11) = 13.656, *p* = .004) deviants.

**MMN Additivity**

Paired-sample *t*-tests were employed to compare the two bi-dimensional MMNs at the same cluster of electrodes. This confirmed that the empirical double deviant MMN was indeed significantly smaller than the predicted MMN, *t*(11) = 4.429, *p* = .001.

To test for the possibility that the 26 ms latency difference between the consonant and pitch MMNs contributed to the absence of additivity, subjects were divided into two groups based on whether they showed significantly later pitch MMN or not at the six chosen electrodes. This created a significant-difference group of 5 subjects (average difference 57ms) and an non-significant-difference group of 7 subjects (average difference 3ms). A 2 (Latency difference: significant & non-significant) × 2 (Mean amplitude: the observed MMN & the predicted MMN) ANOVA was then conducted. Results confirmed the smaller amplitude of the observed double MMN compared with the predicted one, *F*(1, 10) = 17.693, *p* = .002. There was no significant group effect, *F*(1, 10) = .662, *p* = .435. Importantly, the amplitude × group interaction was also not significant, *F*(1, 10) = 1.054, *p* = .392, indicating that the latency difference between the consonant and pitch MMNs did not modulate the degree of under-additivity and therefore could not serve as an explanation for our finding.
