# Supplementary figures and images for: Integration of Consonant and Pitch Processing as Revealed by the Absence of Additivity in Mismatch Negativity
Source: PLoS One. 2012 May 31;7(5):e38289. doi: 10.1371/journal.pone.0038289 (PMC3365020; doi:10.1371/journal.pone.0038289)

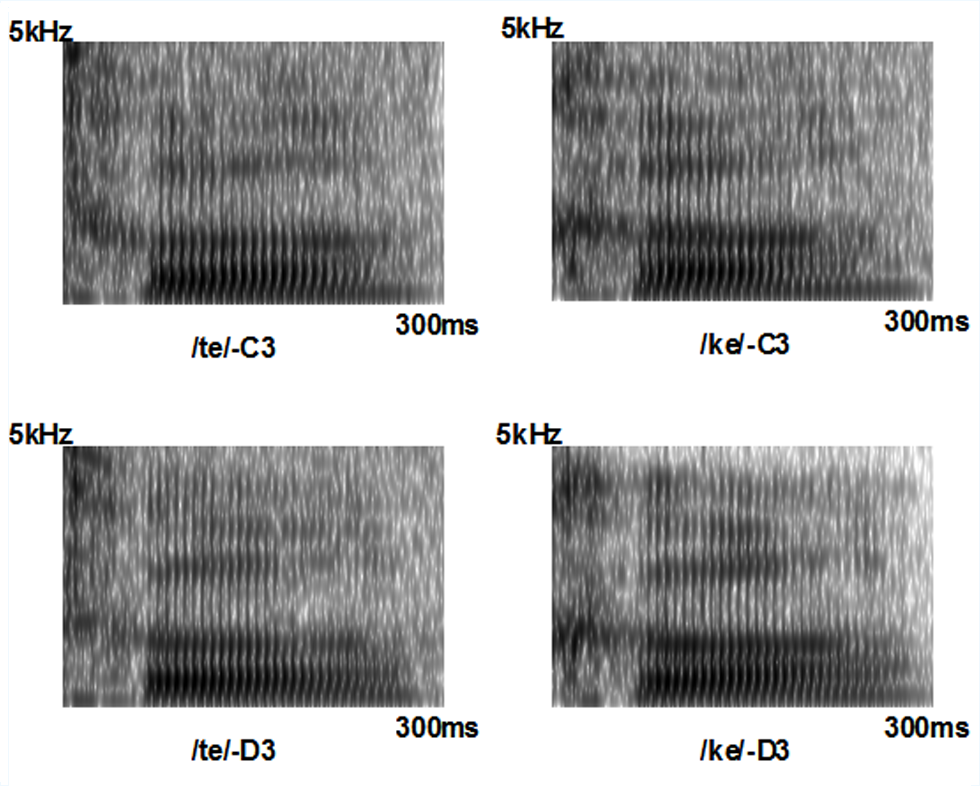

Supplement: Figure S1 — Spectrograms of the four auditory stimuli. These sounds were Chinese initial consonants /t/ and /k/ (actually pronounced as consonant-vowel syllables /te/ and /ke/ when alone) sung at two pitches (C3 = 130 Hz and D3 = 146 Hz) with a duration of 300 ms. (TIF) [file pone.0038289.s002.tif]

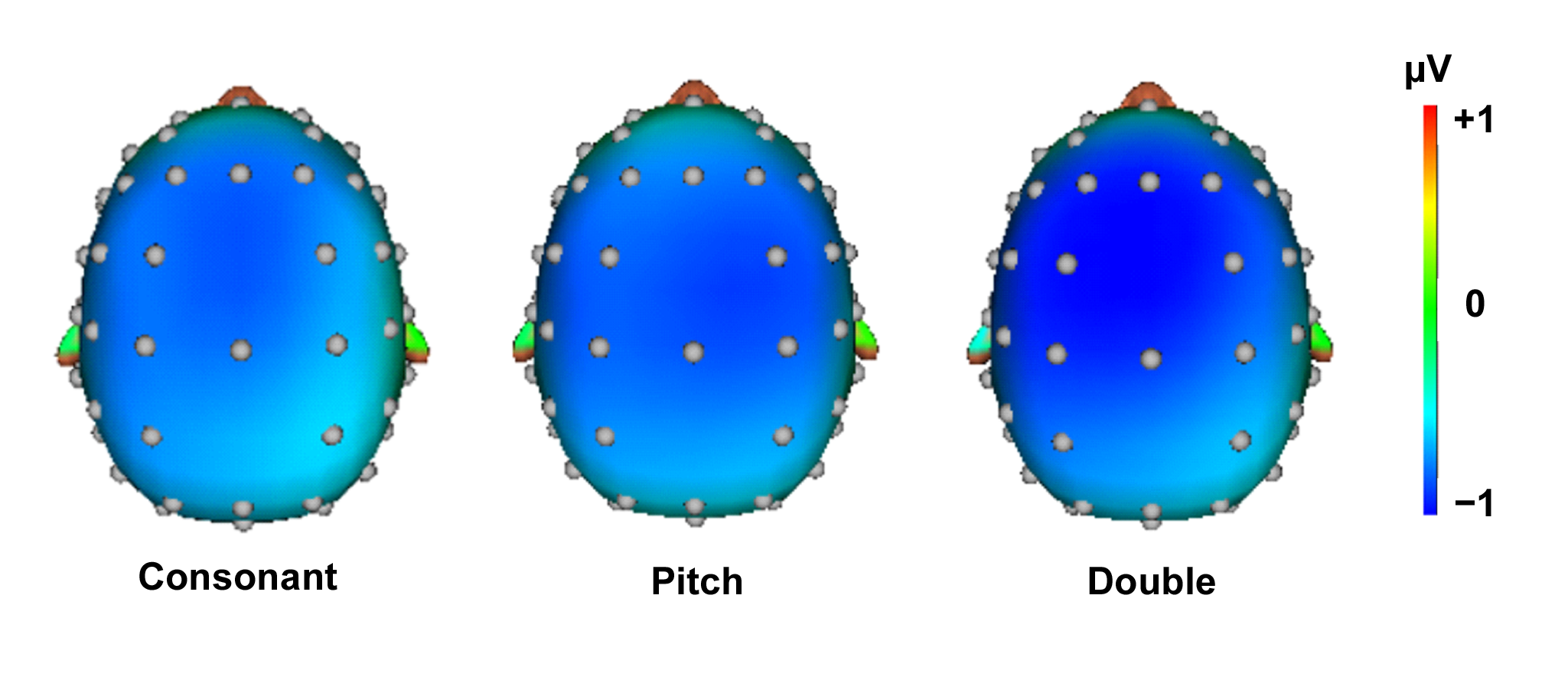

Supplement: Figure S2 — The average scalp topographies of the MMN in each condition. The mean amplitudes of the MMNs were calculated over a 80-ms window centered at the peak. No significant difference or hemispheric dominance was found in the scalp distributions of the MMNs to consonant, pitch, and double deviants. (TIF) [file pone.0038289.s003.tif]
